# Microanchored borehole fiber optics allows strain profiling of the shallow subsurface

**DOI:** 10.1038/s41598-021-88526-8

**Published:** 2021-04-28

**Authors:** Cheng-Cheng Zhang, Bin Shi, Song Zhang, Kai Gu, Su-Ping Liu, Xu-Long Gong, Guang-Qing Wei

**Affiliations:** 1 School of Earth Sciences and Engineering, Nanjing University, Nanjing, 210023 Jiangsu China; 2 Yuxiu Postdoctoral Institute, Nanjing University, Nanjing, 210023 Jiangsu China; 3 Nanjing University High-Tech Institute at Suzhou, Suzhou, 215123 Jiangsu China; 4 Key Laboratory of Earth Fissures Geological Disaster, Ministry of Natural Resources, Geological Survey of Jiangsu Province, Nanjing, 210080 Jiangsu China; 5 Suzhou NanZee Sensing Technology Ltd., Suzhou, 215123 Jiangsu China

**Keywords:** Natural hazards, Solid Earth sciences, Engineering, Fibre optics and optical communications

## Abstract

Vertical deformation profiles of subterranean geological formations are conventionally measured by borehole extensometry. Distributed strain sensing (DSS) paired with fiber-optic cables installed in the ground opens up possibilities for acquiring high-resolution static and quasistatic strain profiles of deforming strata, but it is currently limited by reduced data quality due to complicated patterns of interaction between the buried cables and their surroundings, especially in upper soil layers under low confining pressures. Extending recent DSS studies, we present an improved approach using microanchored fiber-optic cables—designed to optimize ground-to-cable coupling at the near surface—for strain determination along entire lengths of vertical boreholes. We proposed a novel criterion for soil–cable coupling evaluation based on the geotechnical bearing capacity theory. We applied this enhanced methodology to monitor groundwater-related vertical motions in both laboratory and field experiments. Corroborating extensometer recordings, acquired simultaneously, validated fiber optically determined displacements, suggesting microanchored DSS as an improved means for detecting and monitoring shallow subsurface strain profiles.

## Introduction

Shallow geohazards, such as landslides, debris flows, ground subsidence, and sinkhole collapses, can have devastating effects on populations, economies, and landscapes across the world. The initiation and evolution of these near-surface hazards are often accompanied by measurable deformation^1–3^, and therefore measuring and monitoring their spatio-temporal displacements is essential to implementing early warning systems. Of the methods for vertical deformation acquisition, interferometric synthetic aperture radar (InSAR) and global navigation satellite system (GNSS) are commonly used to detect land-surface elevation changes^4^. These ground-based or remotely sensed techniques have proved to be effective in mapping large-scale ground motions^5^, but they do not allow for subsurface deformation profiles to be obtained. Drilling is a common means to determine lithology; by installing extensometers in drilled boreholes, deformations occurring at certain depths below the ground surface can be observed^6^. While highly precise measurements can be made using borehole extensometry, the spatial resolution for such systems is often constrained by discretely instrumented “measuring points”—markers deployed commonly at depths corresponding to critical layers.

Fiber-optic sensing has advanced significantly in the past few years for strain determination in many areas of earth science and engineering^7–14^. Fiber-optic sensing technologies are normally categorized according to the measurand or the optical scattering mechanism whereby the measurement is made^15,16^. The fiber sensing method utilized for static strain detection is often referred to as distributed strain sensing (DSS) while for dynamic strain acquisition as distributed acoustic/vibration sensing (DAS/DVS)^17^. An attractive feature of the broad category of fiber-optic sensing technologies is their ability to make spatially continuous strain (or strain-rate) recordings along a fiber-optic cable up to tens of kilometers in aperture. This advantage has been instrumental, for example, in localizing accurately active compaction zones resulting from subsurface resources exploitation^18–20^ and better characterizing hydromechanical responses^21,22^.

The mechanical coupling between fiber-optic cables and Earth, depending on both cable construction and installation^17^, is an important influencing factor to carrying out successful fiber-optic monitoring campaigns. Many have reported that the quality of fiber-optic data, for either DSS^7,23–25^ or DAS^26^, is strongly conditioned by the degree of rigid ground–cable coupling (hereafter we will focus on DSS to limit this study’s extent). This is especially the case when the deformation of low-confined upper layers is of particular interest, and can be exacerbated by highly saturated weak strata such as those containing large amounts of soft soils. In this respect, correction of measured strains via rigorous ground-to-fiber strain transfer analysis has been proposed to be a potential solution^27^, but it would be better for field applications to have enhanced fiber-optic instrumentation, such as a specialty cable that can be rigidly coupled to its surroundings.

Using anchors to improve interface bonding between reinforcements and surroundings is a common practice in geotechnical engineering^28,29^. This has inspired the DSS community to attach anchor-like elements mechanically to outer coatings or jackets of fiber-optic cables, forming dedicated cables capable of detecting displacements of laboratory physical models^30–33^ or in a field setting via horizontally-trenched direct burial^34^. Pullout tests and shear zone simulation tests were performed to confirm the performance of a shallowly trenched, three-dimensional microanchored cable for landslide monitoring^35^. As to theory, the interaction of tube-anchored cables with surrounding soils has been interpreted from the perspective of interface shearing^36,37^, extending the framework developed primarily for unanchored DSS^38^. While this allows the overall interface shear strength between soil and anchored cables to be estimated, it precludes the consideration of passive earth pressure effects commonly observed during soil–anchor interaction^29^.

We describe here an improved fiber-optic DSS approach for sensing vertical ground displacements with microanchored strain sensing cables deployed in boreholes. We fabricated three microanchors to enhance soil–cable interlocking effects adding on previous work^37^. We proposed a new criterion for assessing soil–cable coupling based on the geotechnical bearing capacity theory. We examined the effects of confining pressure, soil and interface shear strength parameters, and anchor type and dimension on the performance of the microanchored DSS system. We demonstrated the feasibility of this improved methodology through elementary testing, physical modeling, and a field experiment conducted in a coastal setting.

## DSS measurement principle

**Figure 1:**
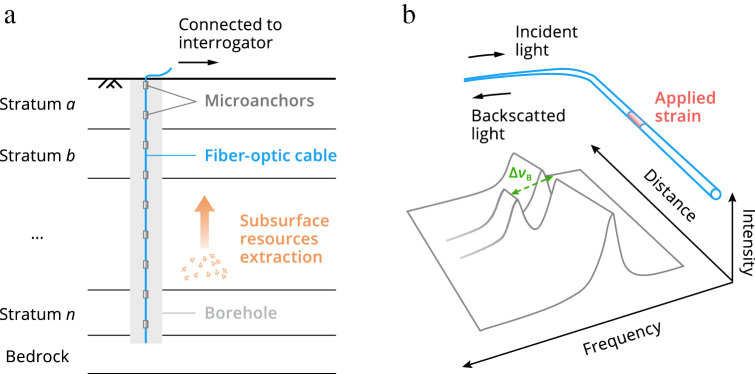
(**a**) Schematic of distributed sensing of stratum deformation resulting from subsurface resources extraction with a borehole-embedded microanchored fiber-optic cable. (**b**) Diagram depicting the measurement principle of fiber-optic distributed strain sensing (DSS). Brillouin optical time-domain reflectometry—requiring the access from only one end of the cable—is shown as an example (See the “DSS measurement principle” section for more Brillouin- or Rayleigh-based DSS techniques). External strains acting on the cable will induce a shift in frequency of backscattered Brillouin light ($\Delta \nu_{{\text{B}}}$), which can be detected by a fiber-optic interrogator. By repeatedly launching light pulses into the fiber, a complete strain profile along the entire borehole length can be determined. Although double-ended approaches (e.g., Brillouin optical time-domain analysis) can generally achieve higher strain accuracy, single-ended deployment is most often the only viable solution in many field applications^17^. Temperature compensation may be performed with a colocated strain-insensitive sensing cable installed in the same borehole.

Figure 1a shows schematically a microanchored fiber-optic cable buried in a borehole for the detection of vertical displacements of geological formations resulting from subsurface resources extraction. DSS techniques used for fiber strain acquisition are based on Brillouin or Rayleigh scattering. These include Brillouin optical time-domain reflectometry (BOTDR), Brillouin optical time-/frequency-domain analysis (BOTDA/BOFDA), optical frequency-domain reflectometry (OFDR), and tunable‐wavelength coherent optical time‐domain reflectometry (TW‐COTDR)^15–17^. Taking the BOTDR technique—requiring the access from only one end of the cable—as an example (Fig. 1b), external strains (referred to axial strain if not otherwise stated) acting on the cable will induce a shift in frequency $\Delta \nu_{{\text{B}}}$ of the Brillouin backscattered light inside the fiber detectable by a BOTDR interrogator. The strain change $\Delta \varepsilon$ can be determined according to^15^:

1 $$ \Delta \varepsilon = \frac{1}{{C_{{\text{e}}} }}(\Delta \nu_{{\text{B}}} - C_{{\text{T}}} \Delta T) $$

where $C_{{\text{e}}}$ is the frequency shift–strain coefficient, $C_{{\text{T}}}$ is the frequency shift–temperature coefficient, and $\Delta T$ is the change in temperature that can be quantified using a colocated temperature sensing cable insensitive to mechanical strains. Because Brillouin backscattering is generated at each point of the fiber, by repeatedly launching light pulses into the fiber a complete strain profile of the deforming strata along the entire borehole length can be mapped. Note that although double-ended approaches (e.g., BOTDA) can generally achieve higher strain accuracy, single-ended deployment is most often the only viable solution in many field applications (e.g., borehole strain profiling)^17^.

Durability is a central concern for any instrument installed in subsurface environments. Theoretically, borehole-embedded fiber-optic DSS systems can be permanently used for deformation observation as fiber-optics are inherently corrosion resistant. In practice, however, fiber coatings or jackets will degrade with time and fibers may break due to large deformation (the ultimate tensile strain of fiber-optics is ~ 2%, i.e., 20,000 με). Our first borehole DSS system was deployed in Shengze (Southern Yangtze Delta, China) in 2012^24^, and strain acquisition has been performed routinely for nearly ten years. We anticipate such systems would survive and function properly for at least several decades; a robust yet strain-sensitive cable is crucial.

## Fabrication of microanchored cables

Anchor-like elements are viewed as essential to ensuring sufficient ground–cable coupling and hence the DSS measurement quality can be improved^35^. For this purpose, we fabricated three types of microanchors—disc, cylinder, and spindle. These anchors were attached at discrete points to commercially available fiber-optic strain sensing cables using epoxy resin adhesives. In doing so, three dedicated cables were developed, covering both field and laboratory application scenarios; their features and properties are summarized in Table 1. The disc-anchored cable is well suited for low-confined laboratory physical modeling, as the 0.9-mm- or 2-mm-diameter thermoplastic polyurethane (TPU)-jacketed cable (NZS-DSS-C07 by NanZee Sensing Ltd.) can readily be integrated into loose media, owing to its relatively low Young’s modulus (*E* =  ~ 1 GPa), and the discs can enhance considerably soil–cable interlocking effects. The cylinder- or spindle-anchored cable utilizes a 5-mm-diameter steel strand-reinforced, polyethylene (PE)-jacketed cable (NZS-DSS-C02; *E* =  ~ 8 GPa). This ensures high survival rates during sensor deployment. Moreover, the small-diameter cylinders and spindles—compared to discs—render the fabricated cables suitable for direct burial installations in field monitoring boreholes.

**Table 1:**
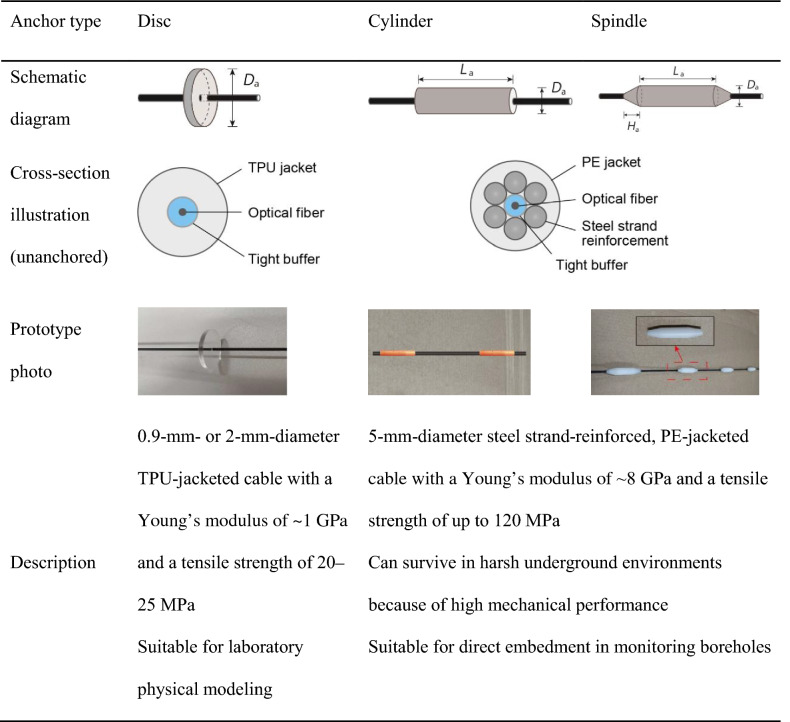
Three microanchored fiber-optic cables developed for deformation sensing in the near surface environment.

The optical fiber depicted in the cross-section illustration is comprised of a fiber core (silica core + cladding) and a coating. The unanchored strain sensing cables are commercially available (NanZee Sensing Ltd.): the TPU-jacketed (NZS-DSS-C07); the PE-jacketed (NZS-DSS-C02). Note that no anchor–cable interface debonding was found in any of the applications presented. TPU = thermoplastic polyurethane; PE = polyethylene. Refer to Ref.^35^ for a cable with special three-dimensional “dead” anchors suitable especially for detection of shear deformation such as a creeping landslide.

## Interaction mechanism between soil and microanchored cable

### Pullout resistance mechanism of bearing microanchored cable

**Figure 2:**
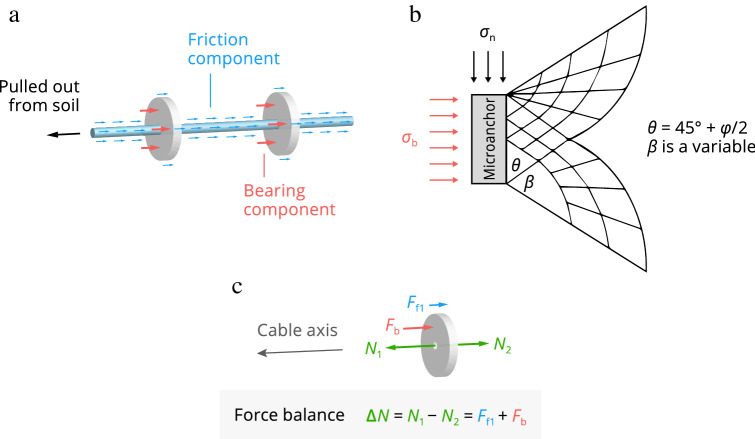
Interaction between soil and microanchored fiber-optic cable, illustrated with an example of disc-shaped microanchors. (**a**) Resistive force components for cable pulled out from soil. (**b**) Modified punching shear failure mechanism of microanchor (after ref^29^). (**c**) Force diagram of a single microanchor.

We first elaborated on the interaction mechanism between soil and a buried microanchored cable through a concise theoretical analysis (Fig. 2), which is a first step toward the successful application of the proposed methodology. The analysis builds on the bearing capacity theory presented by Jewell^28^ and Bergado et al.^29^, originating from geotechnical engineering.

During pullout, the resistance of the microanchored cable is composed mainly of two parts (Fig. 2a): the frictional force component caused by sliding between the cable surface and soil, and the bearing capacity component generated by extrusion between the microanchors and soil. Hence, the ultimate pullout resistance $F_{{\text{r}}}$ of the microanchored cable can be expressed by:

2 $$ F_{{\text{r}}} = F_{{{\text{fr}}}} + F_{{{\text{br}}}} $$

where $F_{{{\text{fr}}}}$ is the soil–cable interface friction resistance that can be determined according to the Mohr–Coulomb theory, and $F_{{{\text{br}}}}$ is the bearing resistance of microanchors. Note that $F_{{{\text{fr}}}}$ may be further divided into the friction resistance between the soil and the anchor $F_{{{\text{fr1}}}}$ and that between the soil and the unanchored cable segment $F_{{{\text{fr2}}}}$.

The microanchor bearing resistance $F_{{{\text{br}}}}$ can be evaluated as follows:

3 $$ F_{{{\text{br}}}} = \frac{{L_{{\text{c}}} }}{{L_{{\text{s}}} }}S\sigma_{{\text{b}}} $$

where $L_{{\text{c}}}$ is the embedment length of the microanchored cable; $L_{{\text{s}}}$ is the spacing between the microanchors; $S$ is the surface area of the microanchor; and $\sigma_{{\text{b}}}$ is the bearing stress of a single microanchor that can be evaluated by:

4 $$ \sigma_{{\text{b}}} = \sigma_{{\text{n}}} N_{{\text{q}}} + cN_{{\text{c}}} $$

where $\sigma_{{\text{n}}}$ is the applied stress normal to the cable axis; $c$ is the soil cohesion; and $N_{{\text{q}}}$ and $N_{{\text{c}}}$ are the bearing capacity factors associated with the bearing failure mode.

Existing pullout bearing failure mechanisms include the general shear failure, punching shear failure, and modified punching shear failure. Among the three failure modes, the general and punching shear failures form the upper and lower bounds of the problem, while the modified punching failure can well describe the bearing failure characteristics of grid reinforcements such as geogrids and geotextiles^29^. Hence, the modified punching failure mode was employed herein to describe the bearing mechanism of microanchored fiber-optic cables, and $N_{{\text{q}}}$ and $N_{{\text{c}}}$ can be respectively expressed as:

5 $$ N_{{\text{q}}} = \left[ {\frac{1 + k}{2} + \frac{1 - k}{2}\sin (2\beta - \phi )} \right]\frac{1}{\cos \phi }e^{2\beta \tan \phi } \tan \left( {\frac{{\uppi }}{4} + \frac{\phi }{2}} \right) $$

6 $$ N_{{\text{c}}} = \frac{1}{\sin \phi }e^{2\beta \tan \phi } \tan \left( {\frac{{\uppi }}{4} + \frac{\phi }{2}} \right) - \cot \phi $$

where $\phi$ is the soil internal friction angle; $k$ is the lateral earth pressure coefficient; and $\beta$ is the angle of the rotational failure zone (Fig. 2b). For $k$ = 1 and $\beta$ = π/2, theoretical predictions were found to agree well with laboratory test data^29^, and $N_{{\text{q}}}$ and $N_{{\text{c}}}$ are thus reduced to:

7 $$ N_{{\text{q}}} = \frac{1}{\cos \phi }e^{{{\uppi }\tan \phi }} \tan \left( {\frac{{\uppi }}{4} + \frac{\phi }{2}} \right) $$

8 $$ N_{{\text{c}}} = \frac{1}{\sin \phi }e^{{{\uppi }\tan \phi }} \tan \left( {\frac{{\uppi }}{4} + \frac{\phi }{2}} \right) - \cot \phi $$

### Validation of bearing resistance equations via laboratory pullout testing

To explore whether the bearing capacity theory is suitable for describing cable anchor failure, we performed laboratory pullout tests on disc-anchored fiber-optic cables at variable anchor diameters. The setup of the pullout tests is sketched in Supplementary Fig. S1a. The soil used was a poorly graded medium sand. Its physical property parameters are: *G*_s_ = 2.65, *d*_10_ = 0.140 mm, *d*_60_ = 0.472, *C*_u_ = 3.371, *C*_c_ = 1.144, *ρ*_dmax_ = 1.82 Mg m^−3^, and *w*_opt_ = 7.82%. Four anchor diameters were investigated: 10, 20, 30, and 40 mm (Fig. S1b,c). For each test, a microanchored cable was buried in the testing soil at a density of 1.70 Mg m^−3^ in the 500 mm × 160 mm × 160 mm chamber, and was pulled out at a velocity of 0.05 mm s^−1^ while recording pullout forces (± 0.1 N). The test was terminated when pullout failure occurred. As the tests lasted for only one hour, the variation of room temperature was negligible and temperature compensation was thus not necessary.

**Figure 3:**
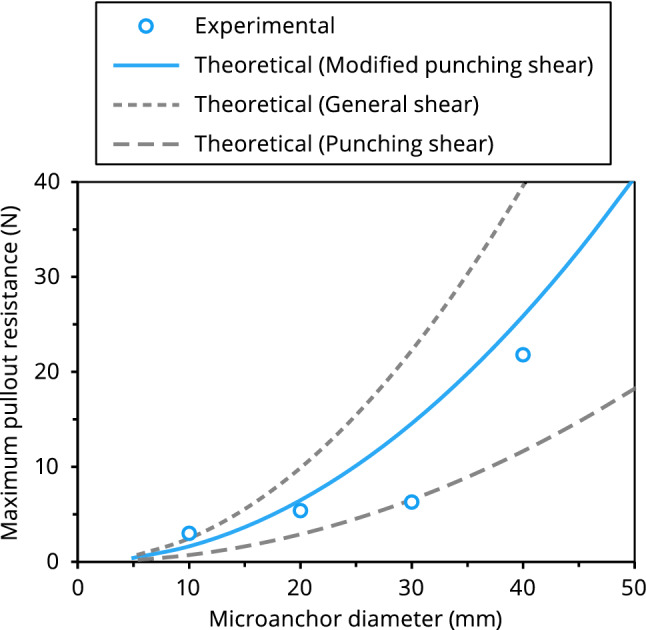
Comparison between experimental and theoretical maximum pullout resistances at varying diameters of disc-shaped microanchor. In addition to modified punching shear failure adopted in this study for describing microanchor bearing failure, upper- and lower-bound values constrained from general and punching shear failure mechanisms are also depicted. The input parameters for theoretical modeling are: $\sigma_{{\text{n}}}$ = 1.36 kPa; $c$ = 0; $\phi$ = 30°; $L_{{\text{c}}}$ = 0.5 m; $D_{{\text{c}}}$ = 0.002 m; and $D_{{\text{a}}}$ = 5–50 mm.

A comparison between the measured pullout resistances and those predicted using the bearing resistance theory (Eqs. (2)–(8)) was carried out; the results are depicted in Fig. 3. Note that in addition to modified punching shear failure, upper- and lower-bound values constrained from general and punching shear failure mechanisms were also computed. The parameters used for theoretical modeling are shown in the caption of Fig. 3. It can be observed that the modified punching shear failure mechanism presently used can better describe the bearing failure behavior of disc-anchored cables compared to the general or punching shear failure. Although these results verified preliminarily the bearing resistance equations, more laboratory testing should be conducted to further validate the proposed method, especially its suitability for describing cylinder- and spindle-anchor cables.

## Criterion for soil–microanchored cable coupling evaluation

### Criterion establishment

Iten et al.^39^ argued that the contact between soil and a buried anchored cable is a combination of overall bonding and point fixation. Experimental evidence^37^ further showed that this combination depends on the deformation stage of the soil–cable interface. Specifically, tube anchors will continue to contribute to the overall interface shear strength after the interface between soil and unanchored segments fails, converting the contact from overall bonding to point fixation. Point fixation may reduce the spatial resolution of DSS^17^, but it is commonly sufficient to obtain a detailed strain profile of subsurface strata. Hence, for ground motion sensing the acquired strain data can be considered as credible provided that the capacity of microanchors has not been reached. In this sense, of particular importance in coupling assessment is the evaluation of stress states of microanchors, especially for those buried in shallow strata. Force equilibrium of a single microanchor yields (Fig. 2c):

9 $$ F_{{\text{a}}} = F_{{{\text{f1}}}} + F_{{\text{b}}} = N_{{1}} - N_{{2}} $$

where $F_{{\text{a}}}$ is the interaction force between the soil and microanchor; $F_{{{\text{f1}}}}$ is the friction force; $F_{{\text{b}}}$ is the bearing force; and $N_{1}$ and $N_{2}$ are the tensions or compressions provided by the unanchored cable segments, which can be calculated using the measured fiber strain:

10 $$ N(x) = \frac{{\uppi }}{4}D_{{\text{c}}}^{2} E_{{\text{c}}} \varepsilon (x) $$

where $D_{{\text{c}}}$ and $E_{{\text{c}}}$ are the diameter and Young’s modulus of the unanchored cable segment, and $\varepsilon (x)$ is the fiber-optic strain measurement.

Combining Eq. (9) with Eq. (10) yields:

11 $$ F_{{\text{a}}} = \frac{{\uppi }}{4}D_{{\text{c}}}^{2} E_{{\text{c}}} \Delta \varepsilon_{{\text{c}}} $$

where $\Delta \varepsilon_{{\text{c}}}$ is the difference in strain measured by the two adjacent unanchored cable segments. Note that if there is no evident step change in strain across the anchors, the strains of the unanchored cable segments may be averaged to obtain $\Delta \varepsilon_{{\text{c}}}$, which is the case for our laboratory and field monitored data.

For the three microanchor types presented in the current work, the ultimate soil–anchor interaction force $F_{{{\text{ar}}}}$ can be readily derived from the bearing capacity theory as:

12 $$ F_{{{\text{ar}}}} = F_{{{\text{fr1}}}} + F_{{{\text{br}}}} = \left\{ \begin{gathered} \frac{{\uppi }}{4}(\sigma_{{\text{n}}} N_{{\text{q}}} + cN_{{\text{c}}} )(D_{{\text{a}}}^{{2}} - D_{{\text{c}}}^{{2}} )\;\;\;\;\;\;\;\;\;\;\;\;\;\;\;\;\;\;\;\;\;\;\;\;\;\;\;\;\;\;\;\;\;\;\;\;\;\;\;\;\;\;\;\;\;\;\;\;{\text{(Disc)}} \\ {\uppi }D_{{\text{a}}} L_{{\text{a}}} \left( {c_{{\text{i}}} + \sigma_{{\text{n}}} \tan \phi_{{\text{i}}} } \right){ + }\frac{{\uppi }}{4}(\sigma_{{\text{n}}} N_{{\text{q}}} + cN_{{\text{c}}} )(D_{{\text{a}}}^{{2}} - D_{{\text{c}}}^{{2}} )\;\;\;\;\;\;\;\;\;\;\;\;\;\;\;{\text{(Cylinder}}) \\ {\uppi }D_{{\text{a}}} (L_{{\text{a}}} { + 2}H_{{\text{a}}} )(c_{{\text{i}}} + \sigma_{{\text{n}}} \tan \phi_{{\text{i}}} ){ + }\frac{{\uppi }}{4}(\sigma_{{\text{n}}} N_{{\text{q}}} + cN_{{\text{c}}} )(D_{{\text{a}}}^{{2}} - D_{{\text{c}}}^{{2}} )\;\;\;\;\;{\text{(Spindle)}} \\ \end{gathered} \right. $$

where $c_{{\text{i}}}$ and $\phi_{{\text{i}}}$ are the cohesion and friction angle of the soil–microanchor interface, and $D_{{\text{a}}}$, $L_{{\text{a}}}$, and $H_{{\text{a}}}$ are the dimensions of the microanchors (Table 1). Note that the side frictional resistance for the disc-shaped microanchor is not included in this formula considering its limited thickness.

When $F_{{\text{a}}}$ is less than $F_{{{\text{ar}}}}$, the ground–cable coupling is sufficient and the fiber optically determined deformation can reflect the true ground motion. Conversely, if $F_{{\text{a}}}$ reaches $F_{{{\text{ar}}}}$, the microanchor fails and the data quality decreases accordingly. This proposed criterion can be used for assessing the reliability of measurements acquired with anchored DSS.

### Toward optimal design of microanchored DSS

To ensure the quality of field monitored fiber-optic strains, a large $F_{{{\text{ar}}}}$ value is desirable. A concise parametric analysis was conducted to investigate the influences of normal stress, microanchor type and dimension, and soil and soil–anchor interface strength parameters on $F_{{{\text{ar}}}}$. The parameters used in the analysis are listed in Supplementary Table S1.

**Figure 4:**
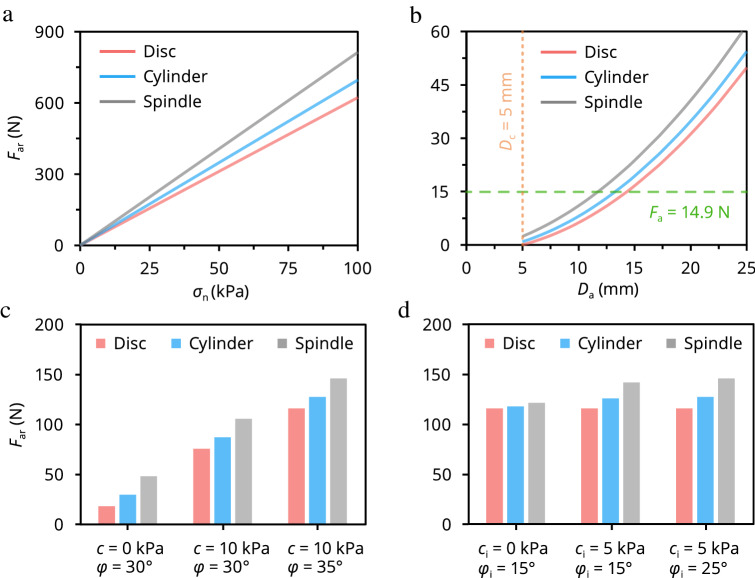
Parametric analysis reveals the effects on the ultimate soil–anchor interaction force $F_{{{\text{ar}}}}$ of variations in model parameters: (**a**) Normal stress $\sigma_{{\text{n}}}$, (**b**) Microanchor diameter $D_{{\text{a}}}$, (**c**) soil strength parameters (*c*, *ϕ*), and (**d**) soil–anchor interface strength parameters (*c*_i_, *ϕ*_i_). $D_{{\text{c}}}$ is the diameter of unanchored cable; $F_{{\text{a}}}$ is the interaction force between soil and microanchor (14.9 N corresponds to a 1% tensile strain). Parameters used in the analysis are summarized in Supplementary Table S1.

It can be observed that $F_{{{\text{ar}}}}$ increased with increasing $\sigma_{{\text{n}}}$ or $D_{{\text{a}}}$, but differed across microanchors (Fig. 4a,b). Because of anchor side friction, the spindle-shaped microanchor had higher $F_{{{\text{ar}}}}$ than the other two microanchors, especially at high $\sigma_{{\text{n}}}$. For field applications, a strain of 1% (corresponding to a $F_{{\text{a}}}$ of 14.9 N under the current parameters) is usually taken as the maximum strain value considering the long-term working performance of the fiber-optic. This strain limit can be used for determining the minimum microanchor diameter required, which is instructive for the design of cable anchors (dashed line, Fig. 4b). The effects of soil and soil–anchor interface strength parameters on $F_{{{\text{ar}}}}$ are illustrated in Fig. 4c,d. $F_{{{\text{ar}}}}$ increased greatly as $c$ or $\phi$ increased; however, the influence of $c_{{\text{i}}}$ and $\phi_{{\text{i}}}$ was comparably insignificant. This is because $N_{{\text{q}}}$ and $N_{{\text{c}}}$ are controlled dominantly by $\phi$ (Eqs. (5) and (6)). These results indicate that ground property parameters need to be considered when designing a microanchored DSS system.

In the following sections we will describe two examples of the application of microanchored DSS: (1) a physical model experiment to investigate the strain response of layered soil under drainage and recharge conditions, and (2) a field experiment to monitor stratum compaction in Yancheng (Jiangsu, China).

## Practical application—I. Laboratory experiment

### Materials and experimental setup

This experiment was performed in a cylindrical box with an internal diameter of 420 mm and a height of 1000 mm (Fig. S2). The model box consists primarily of three segmented plexiglass cylinders with a wall thickness of 10 mm and a height of 300 mm per segment. The bottom of the model box is composed of a square plexiglass plate with a side length of 500 mm and a 100-mm-high plexiglass cylinder (Fig. S3a).

We used a sand as an analogue for the aquifer and a clayey soil for the aquitard. The specific gravity of the sand is 2.65, the internal friction angle is 32°, and the permeability coefficient is 7.71 × 10^–2^ mm s^−1^. The specific gravity of the clayey soil is 2.73, the liquid limit is 34.4%, the plastic limit is 20.0%, and the plastic index is 14.4. Given the low confining pressure present in the model, we chose to use the disc-anchored fiber-optic cable for vertical strain sensing (Fig. S3b). The diameter of the unanchored cable is 1.2 mm with a Young’s modulus of 1.01 GPa. The diameter of the disc is 50 mm, the thickness is 1 mm, and the spacing is 100 mm. An NBX-6050A BOTDA interrogator (Neubrex, Japan; Fig. S3c) was employed to record at a 50 mm sample interval with a 100 mm spatial resolution; the resulting strain accuracy is ± 7.5 με. A settlement gauge was also utilized to measure settlements of soil layers with a measurement range of 0–10 mm and an accuracy of ± 0.01 mm (Fig. S3d).

### Experimental procedure

The physical model was constructed following the procedure described below. Before filling soils in the model box, the microanchored cable was pretentioned (~ 7000 με) and vertically deployed (Fig. S4a). Note that prestrain of the cable allowed compressive deformation to be measured. A 200-mm-thick sand layer, a 300-mm-thick clayey soil layer, and a 100-mm-thick sand layer were then successively compacted in the model box (Figs. S4b,c). The water contents of the sand and clayey soil layers were 18.6% and 16.1%, respectively, whereas the compaction densities were 1.68 Mg m^−3^ and 1.60 Mg m^−3^, respectively. To prevent fine particles from flowing into sand layers, a geotextile was laid at the interface between sand and clayey soil layers (Figs. S4d). Moreover, the settlement gauge was buried at 50 mm depth to measure the total settlement of the 550-mm-thick soil. The two ends of the cable were connected to the BOTDA interrogator to form a U-shaped loop. The constructed model was left for 48 h to allow the cable and surrounding soils to be fully coupled (Figs. S4e). Afterward, it was drained and recharged to investigate the deformation response of the layered soils. The room temperature was controlled at ~ 20 °C during testing.

1. Drainage. First, water was slowly pumped into the box through the inlet on the left side of the model box. After the water level rose to the outlet, the model was left for 24 h to fully saturate the soil layers. Then, remove the water tank and open the water valve at the bottom of the model box. In doing so, the water pressure decreased and the water level dropped gradually, so as to simulate the process of water level decline after groundwater extraction in the field. During this process, fiber-optic strain acquisition and settlement measurements were performed. After the water level and soil strain remained basically stable, the drainage experiment was ended.
2. Recharge. Connect the water valve to the water tank and gradually inject water into the model box. In this process, vertical strains and settlements were also monitored. Similar to drainage, the recharge experiment was stopped after the water level and soil strain were basically stable.

Note that in addition to the experiment described above, an additional experiment having an unanchored cable as the distributed strain sensor was also conducted for comparison purposes.

### Results

**Figure 5:**
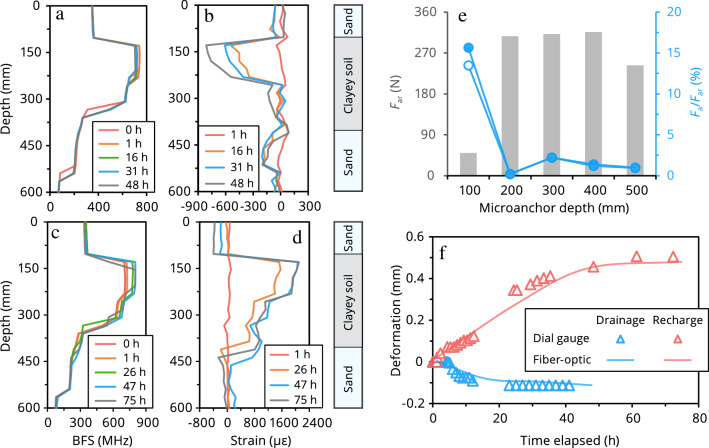
Microanchored fiber-optic DSS applied to a laboratory experiment of layered soil deformation under drainage and recharge conditions. (**a**–**d**) Fiber-optic measurements. (**a**,**b**) Original Brillouin frequency shift (BFS) profiles and derived strain change profiles in the drainage test. (**c**,**d**) Original BFS profiles and derived strain change profiles in the recharge test. (**e**) Calculated ultimate soil–anchor interaction force $F_{{{\text{ar}}}}$ and the degree of mobilization $F_{{\text{a}}} /F_{{{\text{ar}}}}$. Grey bars denote $F_{{{\text{ar}}}}$; blue open circles denote $F_{{\text{a}}} /F_{{{\text{ar}}}}$ (drainage, 48 h); blue solid circles denote $F_{{\text{a}}} /F_{{{\text{ar}}}}$ (recharge, 75 h). (**f**) Fiber optically determined deformation of soil layer at 50–600 mm depth compared with settlement gauge measurements.

Figure 5a–d shows the fiber-optic data measured by the microanchored cable (averaged over the two buried cable segments) at different periods during the drainage experiment. Figure 5a depicts the original Brillouin frequency shifts, which can be converted to strains by multiplying a calibrated frequency shift–strain coefficient. After deducting the initial strain measurements, actual strain change curves were obtained (Fig. 5b). Note that negative (or positive) strains denote compression (respectively, tension). During drainage, the entire soil layer was in a compression state. Compression was especially evident in the upper part of the clayey soil layer (100–250 mm depth), with the maximum negative strain being ~ − 810 με. Figure 5c,d shows soil strain changes during the recharge experiment. It can be observed that the filled soil was basically in a rebound state during the recharge process. The deformation of the bottom sand layer was negligible, whereas the rebound deformation of the middle clayey soil layer was considerably large. Rebound occurred mostly in the first 26 h, and the maximum positive strain reached ~ 2100 με at 75 h.

To determine whether the measured fiber-optic strain data were reliable, we evaluated the stress state of each microanchor along the depth. We analyzed mainly the data corresponding to the largest strain changes (48 and 75 h of drainage and recharge, respectively), where soil–cable decoupling (anchor failure) was most likely to occur. Substituting the basic parameters of the soil layers and microanchor into Eq. (12), the ultimate soil–anchor interaction forces $F_{{{\text{ar}}}}$ for the microanchors were calculated. The computed $F_{{{\text{ar}}}}$ values at depths of 100, 200, 300, 400, and 500 mm were 47.60, 292.34, 296.65, 300.97, and 231.21 N, respectively. According to the original data (Fig. 5a,c), the stress condition of each anchor at the above depths was determined (Fig. 5e). The mobilized interaction forces did not reach their maximum values, indicating that the strain data monitored by the microanchored cable were credible. To further validate the fiber-optic strain measurements, we integrated the measured strains to yield the soil layer deformation at 50–600 mm depth, which was compared with the settlement gauge measurements (Fig. 5f). It can be found that both the trend and magnitude of deformation obtained by the two methods were essentially consistent, thus proving the feasibility of microanchored DSS for monitoring vertical soil deformation at a laboratory scale. Notably, strain profiles measured with the unanchored fiber-optic cable can barely reflect the deformation response of the soil layers due to poor data quality (Fig. S5). This could result from slippage between the soil and the bare cable, owing to insufficient soil–cable coupling in the high soil moisture, low-confined environment. Collectively taken, these results highlight the role of soil–cable interface in soil deformation sensing and underscore the importance of microanchorage in such an unfavorable monitoring environment.

## Practical application—II. Field experiment

### Site description

Yancheng City of Jiangsu Province is located in the eastern coastal region of China, in the middle of the North Jiangsu Plain, and faces the Yellow Sea in the east. The Quaternary sediments in this area, mainly alluvial and marine deposits, were formed under the transportation and accumulation of running water. The shallow strata are composed of loose clay, sub-clay, and medium-fine sand, with a thickness of 200–1600 m.

In recent years, ground subsidence in Yancheng had become more and more serious due to the unreasonable exploitation of subsurface resources and the construction of high-rise buildings^40^. It was reported that the area with a cumulative settlement greater than 200 mm has reached 10.86 km^2^, with the largest settlement being ~ 700 mm. In view of this, we employed the fiber-optic DSS technology to examine the deformation characteristics of subsurface strata and help policy makers cope with the subsidence hazard in the region.

### Monitoring system deployment and data acquisition

In July 2016, a fiber-optic DSS instrumented borehole was constructed in a development zone in Yancheng (33° 21′ 19.38″ N, 120° 10′ 36.39″ E; Fig. S6). The development zone has suffered from severe subsidence because of extensive construction and subsurface mining activities. The monitoring borehole has a depth of ~ 240 m and a diameter of 129 mm. The microanchored fiber-optic cable was deployed in the borehole following the procedure described below.

Drill a vertical borehole in the selected site and perform hole sweeping and washing using clean water. Thread the microanchored cable into the head of a weight guide (Fig. S7a), and wind the cable on a pay-off reel (Fig. S7b). Slowly lower the weight guide and cable into the borehole by controlling the wire rope attached to the cable (Fig. S7c). Backfill the borehole with the prepared fine sand–gravel–bentonite mixture. Keep the cable in a straightened state during this period. Retain the fixator after borehole backfilling and build a monitoring station to achieve long-term deformation sensing.

We installed in this borehole a 5-mm-diameter steel strand-reinforced cable with cylinder-shaped microanchors. The diameter and length of the microanchors are 10 and 90 mm, respectively. The anchor spacing was set to 5 m. The average Young’s modulus of this cable is 8.34 GPa. An AV6419 BOTDR interrogator (CETC-41, China; Fig. S7d) was used for fiber-optic data acquisition with a spatial resolution of 1000 mm and a sample interval of 50 mm; the resulting strain accuracy is ± 50 με. Initial measurements were carried out on December 25, 2016 (used as a baseline), and seven data collections were performed until May 28, 2019.

A group of extensometers were deployed adjacent to the fiber-optic monitoring borehole (~ 5 m apart) by the Geological Survey of Jiangsu Province, at depths of 140, 240, 328, 390, 550, and 590 m. While the extensometers were much deeper than the fiber-optics, their measurements available from November 8, 2017 through May 28, 2019 allowed the fiber optically determined deformation at 0–140, 140–240, and 0–240 m to be corroborated.

### Results

**Figure 6:**
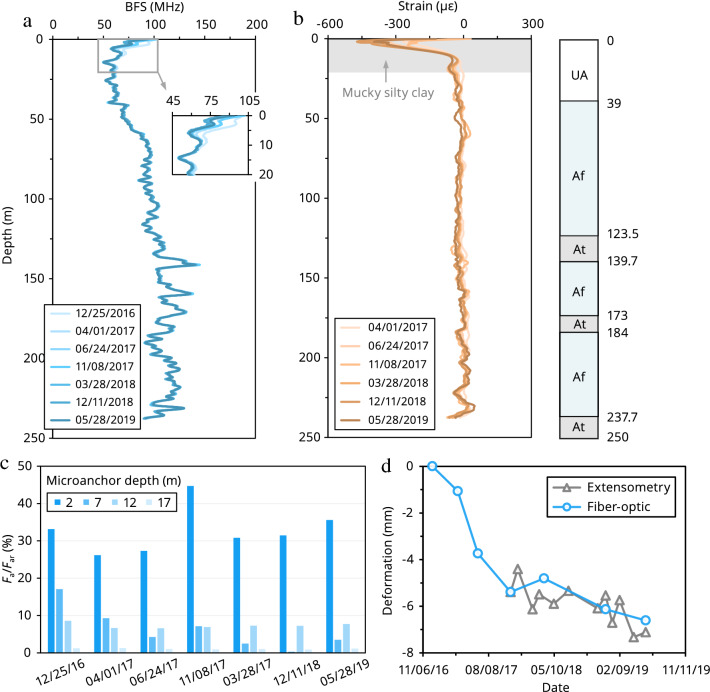
Microanchored fiber-optic DSS applied to a field experiment to monitor subsurface strata deformation in Yancheng (Jiangsu, China). (**a**,**b**) Fiber-optic data acquired with a cylinder-anchored cable in the Yancheng monitoring borehole from December 2016 to May 2019. (**a**) Original Brillouin frequency shift (BFS) profiles. (**b**) Derived strain profiles. UA: unconfined aquifer; Af: confined aquifer; At: aquitard. (**c**) Degree of mobilization of calculated ultimate soil–anchor interaction force $F_{{\text{a}}} /F_{{{\text{ar}}}}$ at different depths. (**d**) Comparison between extensometer measurements and fiber optically determined deformation at 0–240 m depth.

Figure 6a,b depicts the original Brillouin frequency shifts and strain changes measured by the microanchored fiber-optic cable in the Yancheng borehole. It can be observed that compression occurred primarily in the upper 20 m soil layer, with a maximum negative strain of ~  − 400 με. This could be related to the compression of highly compressible mucky silty clays by loading or a variety of civil infrastructures in the development zone.

To evaluate whether the fiber-optic strains were reliable, the stress state of the microanchors at 0–20 m depth was analyzed. Figure 6c shows that with the increase of microanchor depth, the degree of mobilization of soil–anchor interaction force decreased dramatically. This is expected because the ultimate force increased significantly with depth. Although the average value of $F_{{\text{a}}} /F_{{{\text{ar}}}}$ reached approximately 33% for the microanchor at 2 m depth, all these microanchors remained good working condition during the whole process. To further verify the measured fiber-optic data, a comparison between extensometer measurements and fiber optically determined deformation at 0–140, 140–240, and 0–240 m depths was conducted (Fig. 6d, Fig. S8). For 140–240 m depth, because the stratum deformation was relatively small and the control points were limited, there appeared to be some deviations between the two measurements. However, for 0–140 and 0–240 m depths that contained the major compression layer (0–20 m), the two trends agreed with each other. Combined, these results suggest that microanchored DSS could be used for monitoring vertical deformation profiles in a field setting.

## Summary and future work

Pairing DSS with fiber-optic cables installed in vertical boreholes enables the acquisition of spatially continuous strain profiles of subterranean formations. The quality of DSS data is conditioned by ground–cable coupling effects that are difficult to evaluate precisely, especially in near-surface loose sediments under low confining pressures. In this study, we developed an enhanced DSS approach based on improving ground-to-cable coupling conditions using dedicated fiber-optic cables with microanchors attached to their surfaces. We first probed the ground–cable interaction mechanism via theoretical analysis and proposed a bearing capacity-based criterion for data reliability assessment. We then applied the proposed technique to both laboratory and field experiments for the detection of vertical motions. As demonstrated by our results, no buried microanchors failed even at limited confining pressures. We proved the feasibility of microanchored DSS further through comparisons of fiber optically determined deformation with extensometer measurements. We underscore this method’s potential for retrieving high-resolution static and quasistatic strain profiles with a single ground-buried microanchored fiber-optic cable. In particular, the improved quality of strain data acquired in the near surface environment may provide new opportunities for geomechanics and shallow geohazards research. Future studies should aim to achieve higher measurement precision of microanchored DSS via evaluating quantitatively the impact of anchorage on ground-to-fiber strain transfer efficiencies. Moreover, future work to assess the suitability of proposed bearing resistance equations for a variety of microanchor types would allow for a more effective design of anchored DSS systems to detect shallow subsurface displacements.

## Supplementary Information

Supplementary Information.

## Data Availability

The datasets generated during and/or analyzed during the current study are available from the corresponding author on reasonable request.
