# Supplementary material for: Microanchored borehole fiber optics allows strain profiling of the shallow subsurface
Source: Sci Rep. 2021 Apr 28;11:9173. doi: 10.1038/s41598-021-88526-8 (PMC8080621; doi:10.1038/s41598-021-88526-8)
Supplement: Supplementary file 1 — Supplementary Information. [file 41598_2021_88526_MOESM1_ESM.pdf]

Scientific Reports

Supporting Information for

Microanchored borehole fiber optics allows strain profiling of the shallow subsurface

Cheng-Cheng Zhang[1,2,3], Bin Shi[1,*], Song Zhang[1], Kai Gu[1], Su-Ping Liu[1], Xu-Long Gong[4], and Guang-Qing Wei[5]

[1]School of Earth Sciences and Engineering, Nanjing University, Nanjing, Jiangsu 210023, China.

[2]Yuxiu Postdoctoral Institute, Nanjing University, Nanjing, Jiangsu 210023, China.

[3]Nanjing University High-Tech Institute at Suzhou, Suzhou, Jiangsu 215123, China.

[4]Key Laboratory of Earth Fissures Geological Disaster, Ministry of Natural Resources, Geological Survey of Jiangsu Province, Nanjing, Jiangsu 210080, China.

[5]Suzhou NanZee Sensing Technology Ltd., Suzhou, Jiangsu 215123, China.

[*]Correspondence to: shibin@nju.edu.cn (B.S.)

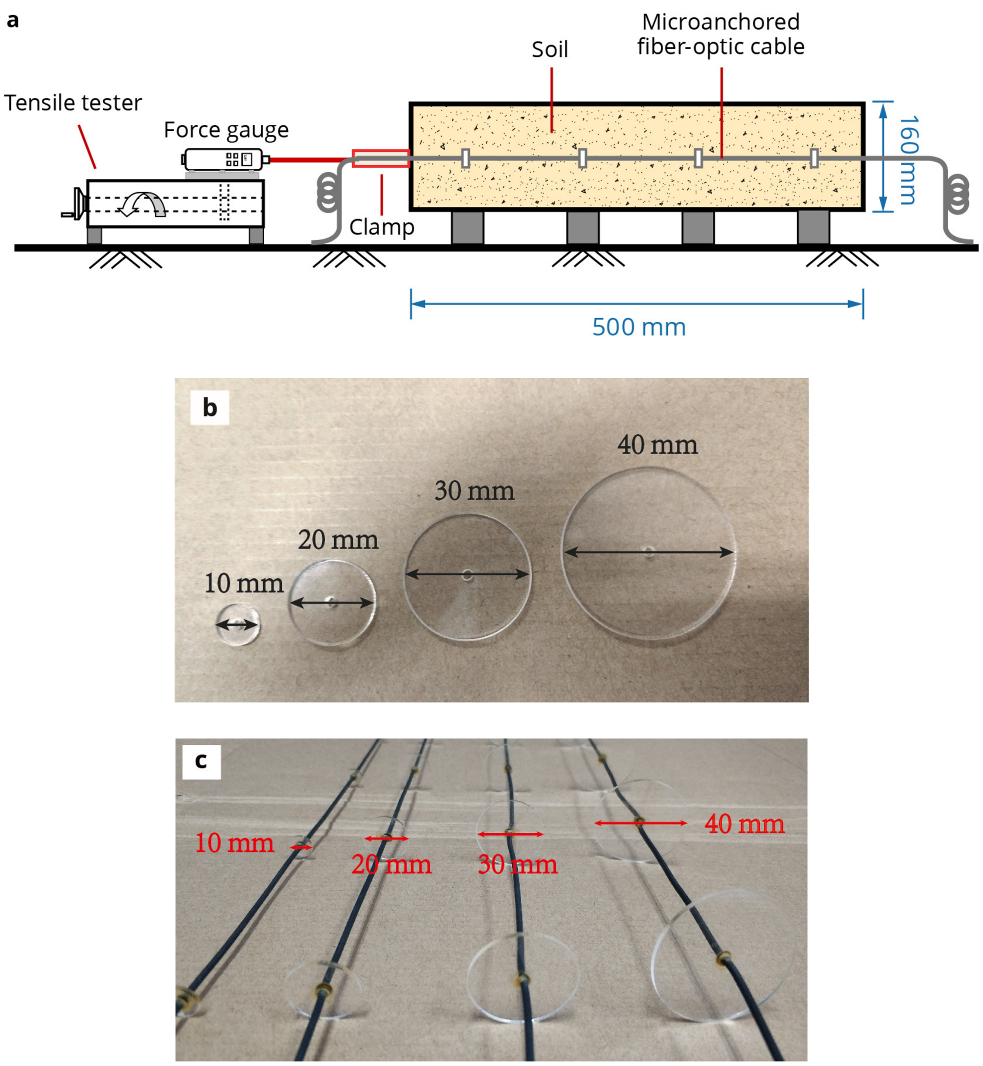

Supplementary Figure S1. (a) Setup of laboratory pullout experiments on disc-anchored fiber-optic cables. **(b)** Photograph of disc-shaped microanchors at four diameters. **(c)** Fabricated cables. The diameter of unanchored cables was 2 mm.

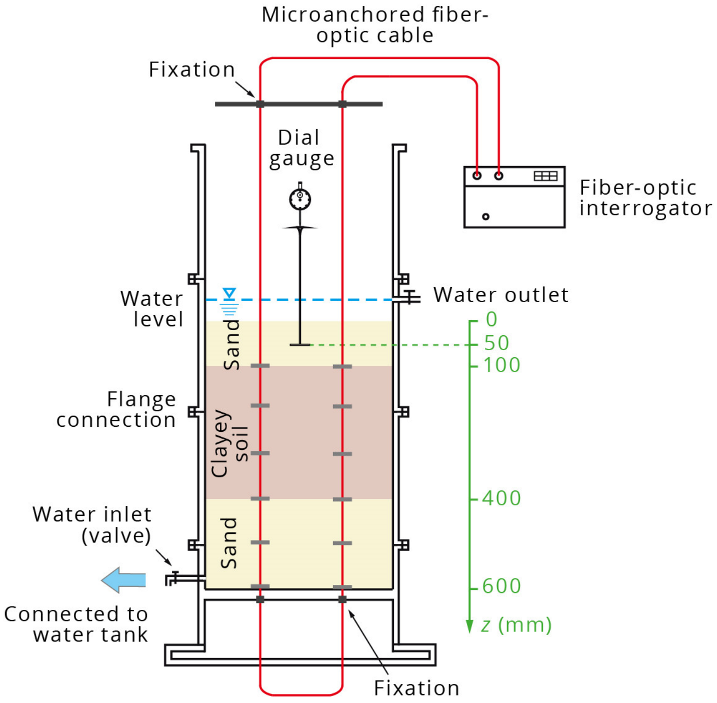

Supplementary Figure S2. Schematic of physical model experiment setup.

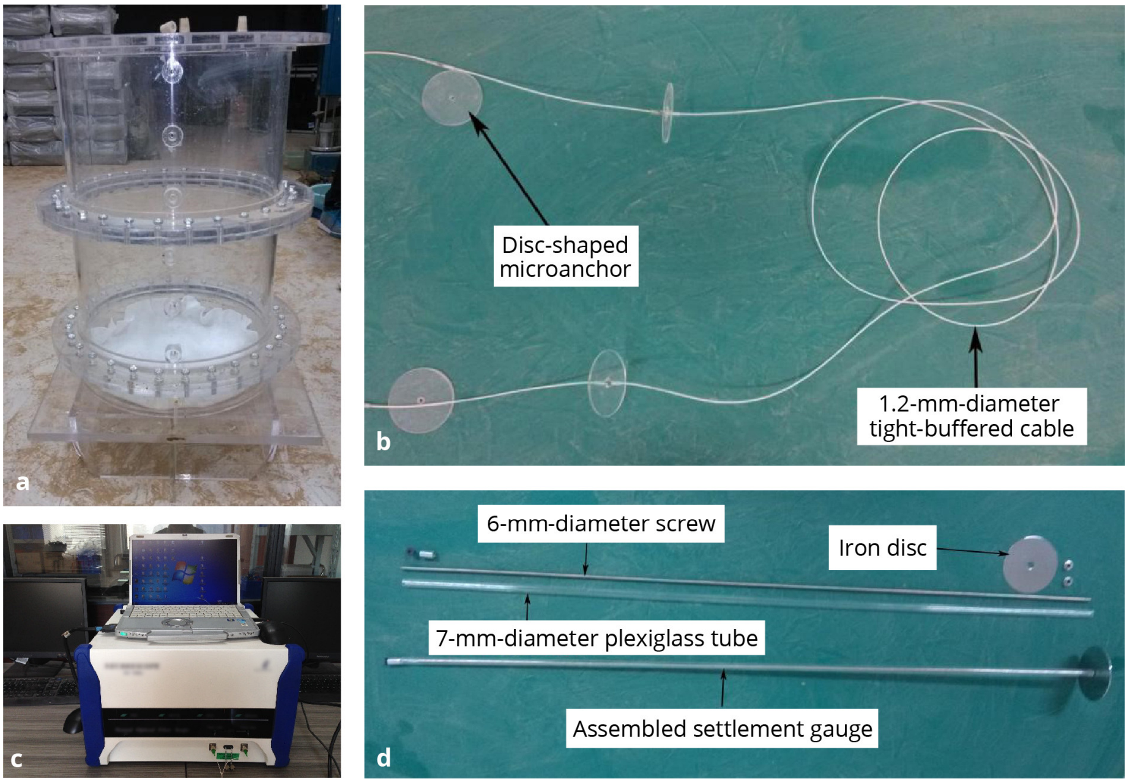

Supplementary Figure S3. Photographs of model box and instruments. **(a)** Segmented plexiglass cylinders. **(b)** Disc-anchored fiber-optic cable. **(c)** NBX-6050A BOTDA interrogator. **(d)** Settlement gauge.

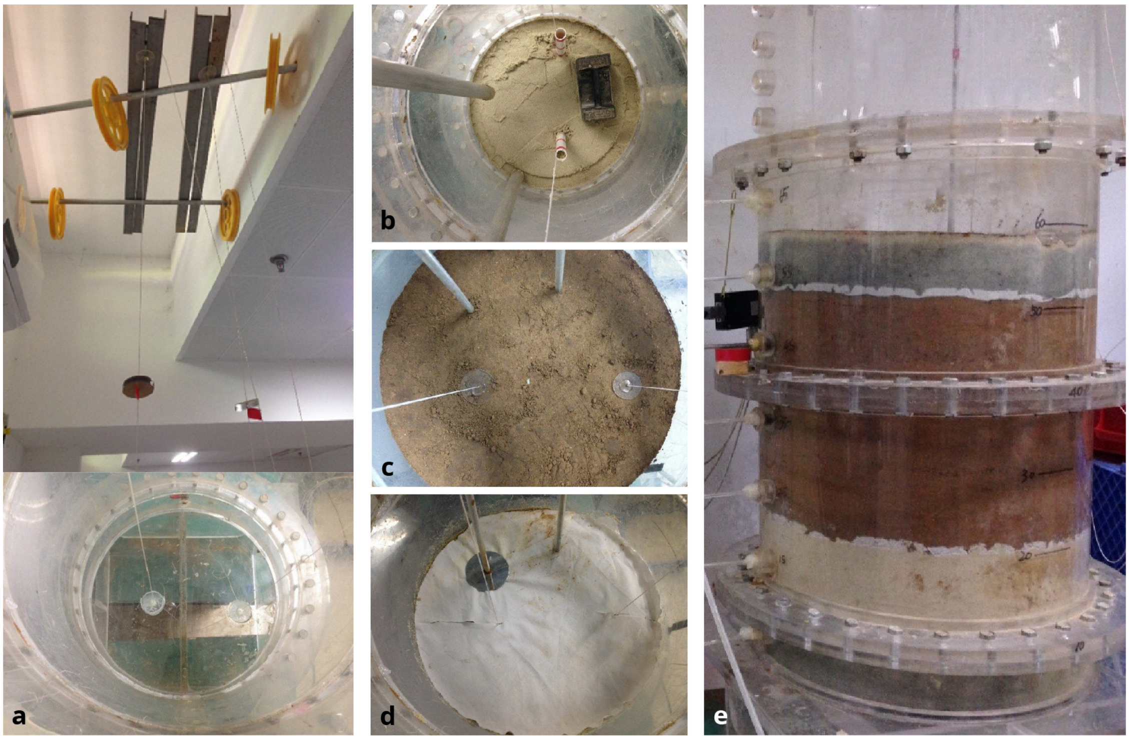

Supplementary Figure S4. Photographs of model preparation. **(a)** Cable pretension and fixation. **(b)** Compacting a sand layer. **(c)** A compacted clayey soil layer. **(d)** A geotextile laid at the interface between sand and clayey soil layers. **(e)** The constructed model left to allow for sufficient soil–cable coupling.

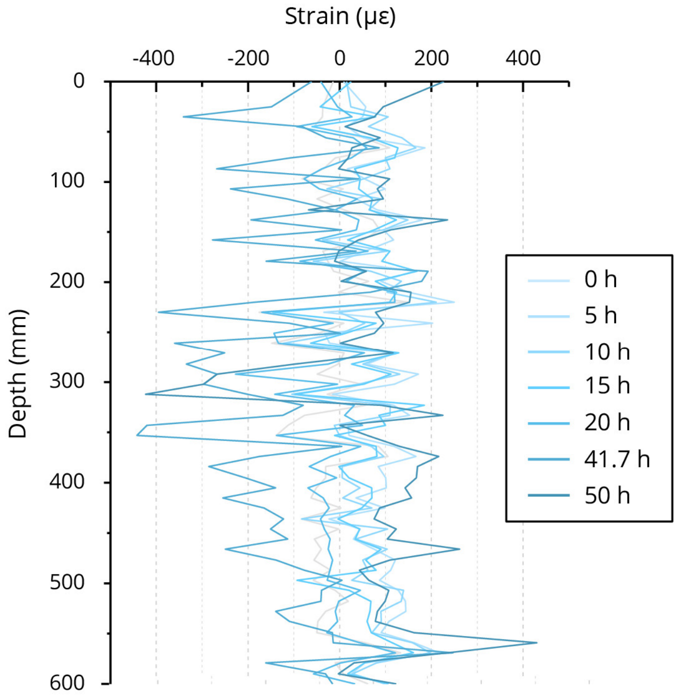

Supplementary Figure S5. Strain profiles measured with an unanchored fiber-optic cable during the drainage experiment. These strain profiles—compared to those acquired with a microanchored fiber-optic cable—can barely reflect the deformation response of the soil layers due to poor data quality, which was attributable to insufficient soil–cable coupling in a high soil moisture, low-confined environment. These results highlight the role of soil–cable interface in soil deformation sensing and underscore the importance of microanchorage in such an unfavorable environment.

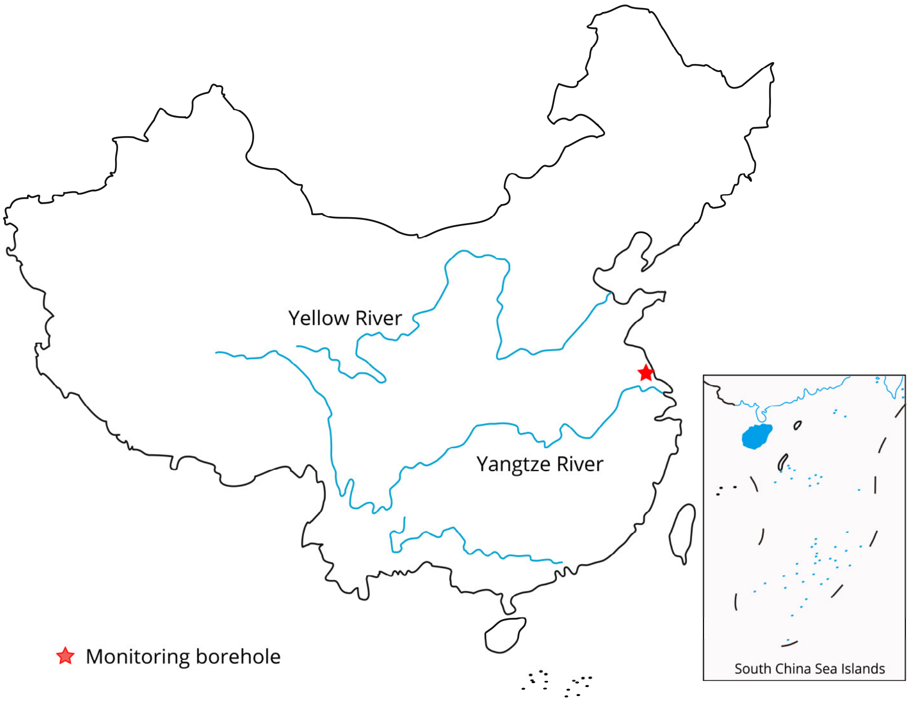

Supplementary Figure S6. Location of field monitoring borehole in Yancheng, Jiangsu Province, China (adapted from ref. S1).

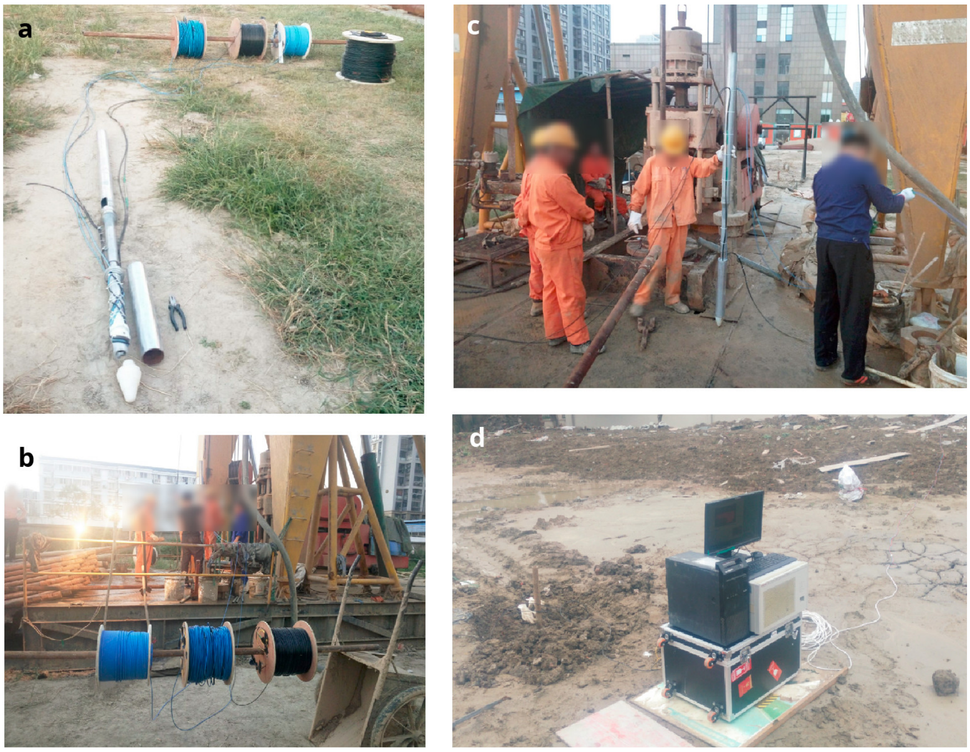

Supplementary Figure S7. Photographs of deployment of micro-anchored fiber-optic cable in the monitoring borehole. **(a)** Threading the cable into the head of a weight guide. **(b)** Winding the cable on a pay-off reel. **(c)** Lowering the cable into the borehole. **(d)** AV6419 BOTDR interrogator.

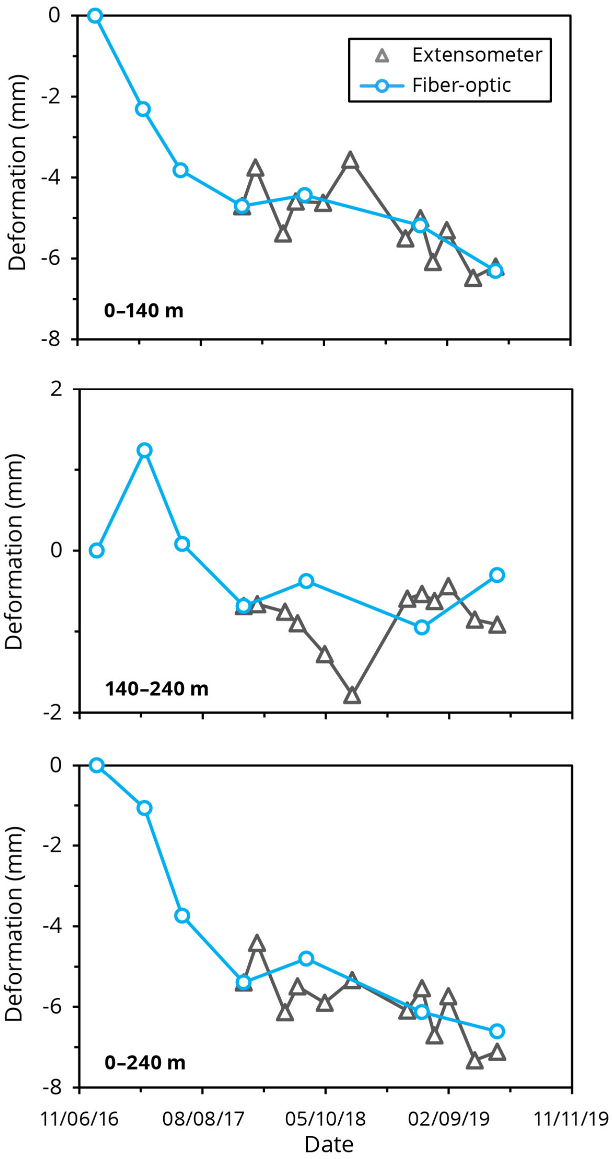

Supplementary Figure S8. Comparison between extensometer measurements and fiber optically determined deformation at 0–140, 140–240, and 0–240 m depths.

Parameter	Value			
	Fig. 4(a)	Fig. 4(b)	Fig. 4(c)	Fig. 4(d)
σ_n (kPa)	0–100	5	5	5
c (kPa)	0	0	(0, 30)<br>(10, 30)<br>(10, 35)	10
ϕ (°)	35	35		35
c_i (kPa)	0	0	5	(0, 15)<br>(5, 15)<br>(5, 25)
ϕ_i (°)	25	25	25	
D_a (mm)	20	5–25	20	20
L_a (mm)	25	25	25	25
H_a (mm)	20	20	20	20
D_c (mm)	5	5	5	5
E_c (GPa)	0.076	0.076	0.076	0.076

Supplementary Table S1. Parameters used for the analysis of the effects of confining pressure, soil and interface strength parameters, and anchor type and dimension on the ultimate anchor–soil interaction force. The analyzed results are shown in Fig. 4 in the main text.

Supplementary Reference

S1. Liu, S.-P. *et al.* Land subsidence monitoring in sinking coastal areas using distributed fiber optic sensing: a case study. *Nat. Hazards* **103**, 3043–3061 (2020).
